# Inhibition of pancreatic EZH2 restores progenitor insulin in T1D donor

**DOI:** 10.1038/s41392-022-01034-7

**Published:** 2022-07-22

**Authors:** Keith Al-Hasani, Ishant Khurana, Lina Mariana, Thomas Loudovaris, Scott Maxwell, K. N. Harikrishnan, Jun Okabe, Mark E. Cooper, Assam El-Osta

**Affiliations:** 1 Department of Diabetes, Central Clinical School, Monash University, Melbourne, Victoria 3004 Australia; 2 Epigenetics in Human Health and Disease Laboratory, Central Clinical School, Monash University, Melbourne, Victoria 3004 Australia; 3 Immunology and Diabetes Unit, St Vincent’s Institute of Medical Research, Fitzroy, Victoria Australia; 4 Department of Clinical Pathology, The University of Melbourne, Parkville, Victoria Australia; 5 Department of Medicine and Therapeutics, The Chinese University of Hong Kong, Sha Tin, Hong Kong SAR; 6 Hong Kong Institute of Diabetes and Obesity, Prince of Wales Hospital, The Chinese University of Hong Kong, 3/F Lui Che Woo Clinical Sciences Building, 30-32 Ngan Shing Street, Sha Tin, Hong Kong SAR; 7 Li Ka Shing Institute of Health Sciences, The Chinese University of Hong Kong, Sha Tin, Hong Kong SAR; 8 Faculty of Health, Department of Technology, Biomedical Laboratory Science, University College Copenhagen, Copenhagen, Denmark

**Keywords:** Preclinical research, Endocrine system and metabolic diseases

## Abstract

Type 1 diabetes (T1D) is an autoimmune disease that selectively destroys insulin-producing β-cells in the pancreas. An unmet need in diabetes management, current therapy is focussed on transplantation. While the reprogramming of progenitor cells into functional insulin-producing β-cells has also been proposed this remains controversial and poorly understood. The challenge is determining why default transcriptional suppression is refractory to exocrine reactivation. After the death of a 13-year-old girl with established insulin-dependent T1D, pancreatic cells were harvested in an effort to restore and understand exocrine competence. The pancreas showed classic silencing of β-cell progenitor genes with barely detectable insulin (*Ins*) transcript. GSK126, a highly selective inhibitor of EZH2 methyltransferase activity influenced H3K27me3 chromatin content and transcriptional control resulting in the expression of core β-cell markers and ductal progenitor genes. GSK126 also reinstated *Ins* gene expression despite absolute β-cell destruction. These studies show the refractory nature of chromatin characterises exocrine suppression influencing β-cell plasticity. Additional regeneration studies are warranted to determine if the approach of this n-of-1 study generalises to a broader T1D population.

## Introduction

T1D is a chronic condition in which the pancreas produces little or no insulin. Even though symptoms usually do not appear before 80% of the β-cell mass has been destroyed, absolute destruction of these cells leads to the dependence on exogenous insulin administration for survival. Two strategies currently exist that focus on replacing the damaged β-cell mass in diabetic patients, involving either whole pancreas or islet transplantation. Although proven to be of clinical utility, these therapies face a real shortage of organ donors together with the associated side-effects of immunosuppressive drugs. Consequently, current research has focussed on the replacement of the lost β-cell and this remains an unmet need for diabetic patients.

Experimental evidence on the inherent plasticity of pancreatic cells has fuelled interest in the regeneration of β-cells. Pancreatic duct cells have been suggested as a source of progenitors for β-cell regeneration.^1^ The master gene involved in endocrine fate determination is Neurogenin 3 (*Ngn3*).^2,3^ *Ngn3* is required for the development of all endocrine cells (α-, β-, δ-, PP- and ε-cells), that are associated with the secretion of specific endocrine hormones within the pancreas. Moreover, during pancreas morphogenesis, *Ngn3* induces the delamination of progenitors from the ductal epithelium through an epithelial-to-mesenchymal transition (EMT) process.^4^ EMT is a key developmental programme by which cells located within an epithelial layer acquire the ability to spread and migrate to a distant site to form new structures mediated by *Sox11*.^5^ In addition to *Ngn3*, *Pdx1* (pancreatic and duodenal homeobox 1) is a key regulator of pancreas development, its expression in progenitor cells driving the differentiation of all exocrine and endocrine pancreatic cells including the genesis of β-cells and the maintenance of β-cell identity.^5^ Furthermore, *Pdx1* is a glucose-responsive regulator of insulin gene expression, where it plays a critical role in the regulation of insulin gene expression.^6^ Thus, *Ngn3* and *Pdx1* appear to be ideal candidates for strategies that aim to influence core transcriptional events using chemical inhibitors, thereby enabling pancreatic β-cell regeneration as a potential path towards improved treatments for T1D.

The objective of this study was to investigate the specific regulation of the H3K27me3 writing enzyme EZH2 that is considered responsible for default suppression (transcriptional gene silencing) critical to the ductal progenitor cell’s developmental programme by enabling them to differentiate into functioning insulin secreting β-cells. The feasibility of this approach has been demonstrated in other contexts. For example, Dal-Pra et al. showed that demethylation of H3K27 is essential for the induction of direct cardiac reprogramming.^7^ An independent group has recently showed similar results, that repression by EZH2-mediated H3K27me3 assists the activation of cardiac genes during human cardiac reprogramming.^8^ We anticipate that our epigenetic approach targeting EZH2 in order to achieve β-cell replenishment and thereby improve glycaemic control could then be applied to relevant clinical settings.

We recently showed that DNA methylation is part of the epigenetic barrier to β-cell differentiation, which underscores the central role of transcriptional regulation in tissue regeneration. When this barrier is removed, endocrine cells are able to differentiate into β-cells. This was a major breakthrough, since a major restriction to repair damaged endocrine tissue in T1D is that the process of β-cell neogenesis from ductal-cell progenitors is limited after birth.^9^ While this was an exciting development, we questioned whether compounds that target corresponding epigenetic pathways in mouse models can be translated to the human context. Despite some of the initial study successes using epigenetic modulators in mice they have shown limited or modest translatability and selectivity in humans. This is likely to be related to complex inter- and intra-cell population differences between mouse and human pancreas.^10,11^

**Fig. 1:**
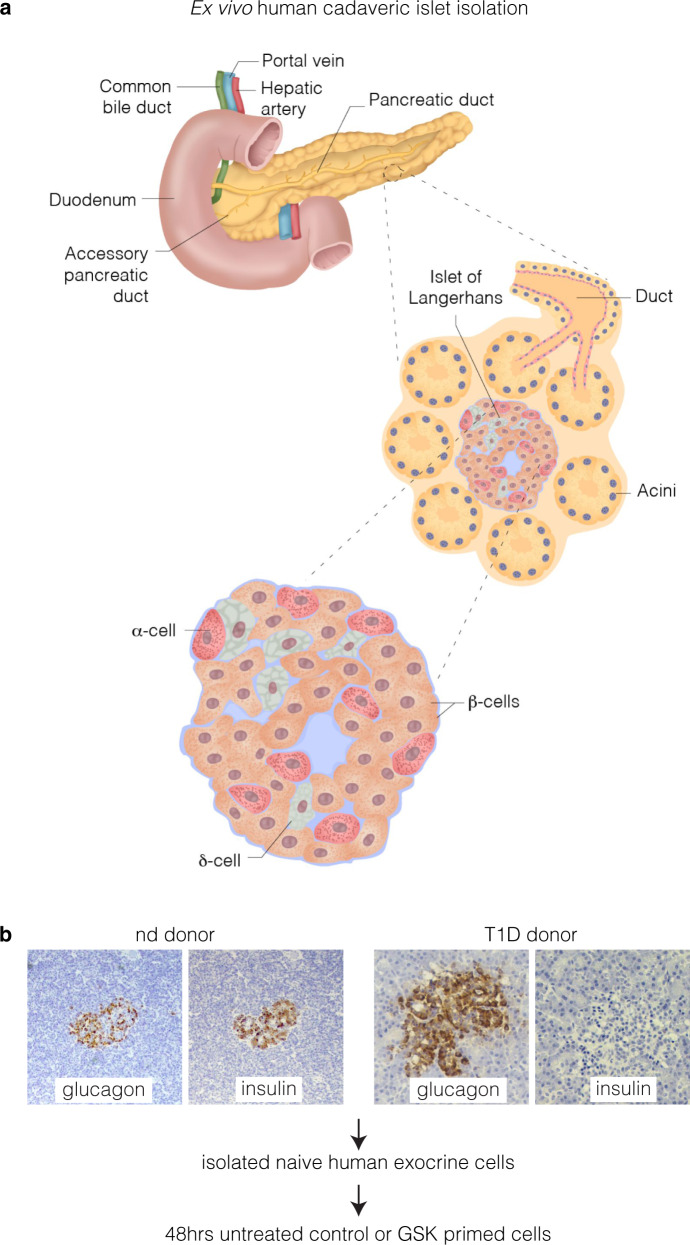
Human cadaveric ex vivo exocrine isolation. **a** Schematic of the human pancreas emphasising the ductal endocrine and exocrine organisation. Pancreatic islet illustrated showing the major cell types. Pancreatic exocrine cells were isolated from cadaveric tissue derived from two non-diabetic and a T1D donor. **b** Representative immunohistochemical insulin and glucagon staining in the non-diabetic donor and T1D donor. Insulin and glucagon expression are indicated by the brown staining in human islets. Note the complete absence of insulin in type 1 diabetic donor

A potential source of β-cells was previously demonstrated with the discovery of α-cell plasticity and the ability of ductal and α-cells to convert into insulin-producing cells.^10,12^ From our studies, we conclusively established the ductal and α-cell ontogeny of these transdifferentiated β-cells by direct lineage tracing experiments. We showed that *Ngn3* re-expression is a feature of pancreatic progenitors in the duct, and *Sox11* is a hallmark of EMT.^13,14^ Equally important was the finding that the α- to β-like cell conversion observed in these models induces the re-expression of *Ngn3* in ductal cells and their differentiation into endocrine cells by reawakening epithelial-mesenchymal-transition.^12,13^ Moreover, we showed recently, that during directed β-cell reprogramming, *Ngn3* and *Sox11* are demethylated.^14^ This finding represents a paradigm shift in our understanding of islet biology and provides new opportunities for future treatment. To our knowledge, there are no published studies that have reported successful induction of exocrine progenitor genes using human pancreatic tissue (Fig. 1a). The rare availability of fresh human pancreas from two non-diabetic adults and a child with T1D (Table 1) prompted us to focus on default exocrine suppression influencing β-cell plasticity.

**Table 1:** Clinical characteristics of type 1 diabetic and non-diabetic donors

	Donor 1	Donor 2	Donor 3
Age	58	42	13
Sex	Male	Female	Female
BMI	28.3	29	18.7
Diabetes	Non-diabetic	Non-diabetic	Type 1
Diabetes duration	N/A	N/A	4.4 years
HbA1c	N/A	N/A	7.7

## Results

**Fig. 2:**
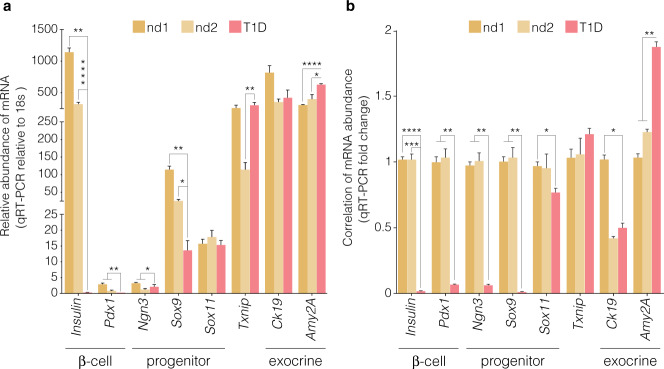
Distinguishable expression of embryonic and β-cell mRNA indices from naïve pancreatic exocrine cells isolated from donors. Abundance of *Ins*, *Pdx1*, *Ngn3*, *Sox9* and *Txnip* mRNA. **a** Expression analyses in naïve exocrine cells showing the comparative abundance of mRNA relative to *18s* assessed by qRT-PCR. Relative expression of mRNA isolated from non-diabetic (nd) and type 1 diabetic (T1D) donors. **b** Correlation of mRNA expression of *Ins*, *Pdx1*, *Ngn3*, *Sox9* and *Txnip* displayed as a function of fold change. Expression analyses of mRNA isolated from non-diabetic (nd) and type 1 diabetic (T1D) donors. Significance is calculated by comparing nd1 v T1D and nd2 v T1D. Student *t* test, *P < 0.05, **P < 0.01, ***P < 0.001, ****P < 0.0001. Error bars represent SEM, *n* = 2

To test our hypothesis that reawakening exocrine and endocrine progenitor cells can be achieved using epigenetic molecules and take on an endocrine cell fate, we examined the effect of the highly selective inhibitor of EZH2 methyltransferase activity, GSK126 on *Nkx6.1, MafA, Pdx1*, *Ngn3*, *Sox11* and *Sox9* using ex vivo human pancreatic tissues from three donors, and the effect of this drug on β-cell regeneration as assessed by an increase in insulin (*Ins*) gene expression. Pancreatic specimens were stained with H&E and immunostained for glucagon and insulin from a non-diabetic and a T1D donor (Fig. 1b). The expression of progenitor and embryogenic markers, *Pdx1, Ngn3*, *Sox9 and Sox11*, as well as specific β-cell markers *Pdx1, Ins, Nkx6.1 and MafA* mRNA levels using qRT-PCR were mirrored in naïve pancreatic exocrine cells using the same non-diabetic and T1D donors (Fig. 2).

**Fig. 3:**
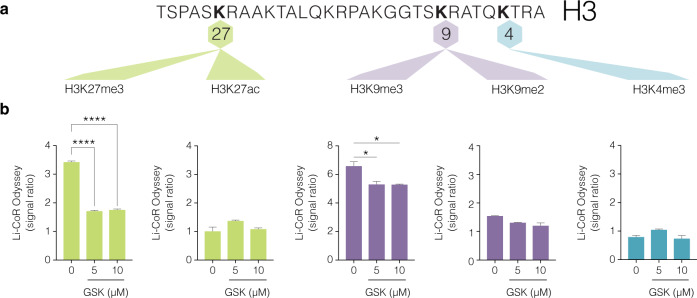
Pharmacological inhibition of EZH2 activity by GSK126 diminishes H3K27me3 and not H3K27ac protein content in human pancreatic ductal cells. **a** Partial histone H3 lysine map of sites for methylation and acetylation. The transcriptionally suppressive marks, H3K27me3, H3K9me3 and H3K9me2 including permissive histone marks, H3K27ac and H3K4me3 are shown. **b** Dose-dependent increase of GSK126 (5 or 10 µM) for 48 h attenuates H3K27me3 in human pancreatic ductal cells. Histones and their associated proteins were prepared by acid extraction. Quantification levels of H3K27me3, H3K27ac, H3K9me2, H3K9me3 and H3K4me3 were calculated and adjusted to overall histone H3 using Li-COR Odyssey. The signal ratio calculated was as follows; H3K27me3/total H3, H3K27ac/total H3, H3K9me3/total H3, H3K9me2/total H3, and H3K4me3/total H3. Vehicle control is DMSO. Ordinary one-way ANOVA was performed on Control vs GSK-126 (*P < 0.05, ****P < 0.0001 error bars are SEM, *n* = 3)

Because GSK126 is a pharmacological inhibitor of EZH2 and trimethylation of lysine (K) at position 27 of histone H3 tail is a powerful regulatory determinant that controls exocrine transcription^15,16^ we assessed H3K27me3 together with the co-repressive H3K9me2 and K3K9me3 marks (Fig. 3a). LiCoR protein analyses confirmed that GSK126 markedly reduced trimethylation at lysine position 27 (H3K27me3) (control vs GSK-126, ****P < 0.0001) and failed to influence histone acetylation (ac) content at the identical lysine position (H3K27ac) in human pancreatic ductal cells (Fig. 3b). Mechanisms of transcriptional suppression mediated by H3K27me3 can cooperate with H3K9 methylation.^17^ Next, we show the transcriptionally suppressive mark H3K9me2 was not altered while H3K9me3 was marginally reduced but not attenuated by GSK126 (control vs GSK-126, *P < 0.05). Furthermore, GSK126 did not influence the transcriptionally permissive histone modification, H3K4me3. This is in accordance with other reports showing that drug-induced reductions in H3K27me3 are effective in re-establishing transcriptional competence.^18^ Taken together, these results suggested H3K27me3 content in human pancreatic ductal cells are influenced by GSK126 to regulate exocrine transcription.

**Fig. 4:**
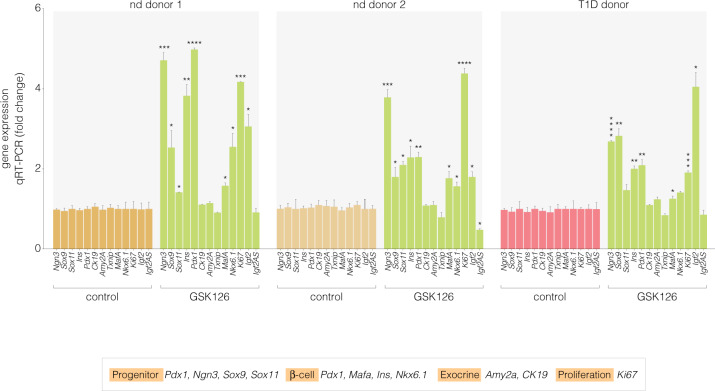
GSK-126 restores the expression of islet indices in non-diabetic and type 1 diabetic pancreatic exocrine cells. Correlation of mRNA abundance of *Ngn3, Sox9, Sox11, Ins, Pdx1, Ck19, Amy2A, Txnip, Mafa, Nkx6.1, Igf2 and Igf2AS* displayed as fold change by qRT-PCR. Significant changes in mRNA abundance were not detected for the ductal marker (Ck19), acinar marker (*Amy2A*) and the glucose-sensor (*Txnip*). Student’s *t*-test was performed on Control vs GSK-126 (*P < 0.05, **P < 0.01, ***P < 0.001, ****P < 0.0001 error bars are SEM, *n* = 3)

In support of our hypothesis, we show that when pancreatic cells are stimulated with the EZH2 inhibitor GSK126, we restore the hallmark genes responsible for the maintenance of pancreatic progenitor identity, *Pdx1*, *Ngn3*, and Sox9^19^ as well as significant expression of the *Ins* gene were observed in the non-diabetic and the T1D donors. Gene expression was assessed by qRT-PCR using amplimers specifically designed to determine human progenitor, β-cell, exocrine, and proliferation markers (Table 2) These results support the notion that EZH2 inhibition restores exocrine multipotency even in chronic disease with total β-cell destruction (Fig. 4). Notably, GSK126 did not significantly alter expression of the acinar marker; *Amy2A*, the ductal marker; *Ck19* or the glucose-sensor; *Txnip*. Of major interest, is the increase in gene expression of the proliferative marker *Ki67*. This was consistent with elevated expression of *Sox9*, a regulator of ductal progenitor cell expansion and differentiation.

**Table 2:** Human primer sequences for mRNA expression

Primer Name	Sequence
NGN3 cDNA F	TCTCTATTCTTTTGCGCCGG
NGN3 cDNA R	CTTGGACAGTGGGCGCAC
SOX9 cDNA F	AGGAAGCTCGCGGACCAGTAC
SOX9 cDNA R	GGTGGTCCTTCTTGTGCTGCAC
INS cDNA F	ACGAGGCTTCTTCTACACACCC
INS cDNA R	TCCACAATGCCACGCTTCTGCA
PDX1 cDNA F	GAAGTCTACCAAAGCTCACGCG
PDX1 cDNA R	GGAACTCCTTCTCCAGCTCTAG
CK19 cDNA F	AGCTAGAGGTGAAGATCCGCGA
CK19 cDNA R	GCAGGACAATCCTGGAGTTCTC
AMY2A cDNA F	GATAATGGGAGCAACCAAGTGGC
AMY2A cDNA R	CAGTATGTGCCAGCAGGAAGAC
TXNIP cDNA F	ACGCTTCTTCTGGAAGACCA
TXNIP cDNA R	CGGTCAAGAAAAGCCTTCAC
MAFA cDNA F	GCTTCAGCAAGGAGGAGGTCAT
MAFA cDNA R	TCTGGAGTTGGCACTTCTCGCT
NKX6.1 cDNA F	CCTATTCGTTGGGGATGACAGAG
NKX6.1 cDNA R	TCTGTCTCCGAGTCCTGCTTCT
SOX11 cDNA F	GCTGAAGGACAGCGAGAAGATC
SOX11 cDNA R	GGGTCCATTTTGGGCTTTTTCCG
Ki67 cDNA F	GAAAGAGTGGCAACCTGCCTTC
Ki67 cDNA R	GCACCAAGTTTTACTACATCTGCC
IGF2 cDNA F	GCAGAGGAGTGTCCGGCAG
IGF2 cDNA R	GGATTCCCATTGGTGTCTGGAA
IGF2 AS cDNA F	CTGCCTAGAGCTCCCTCTTTC
IGF2 AS cDNA R	GCCTGTCCAACAGAAGGGTC
18s cDNA F	CAGCCACCCGAGATTGAGCA
18s cDNA R	TAGTAGCGACGGGCGGTGTG

**Fig. 5:**
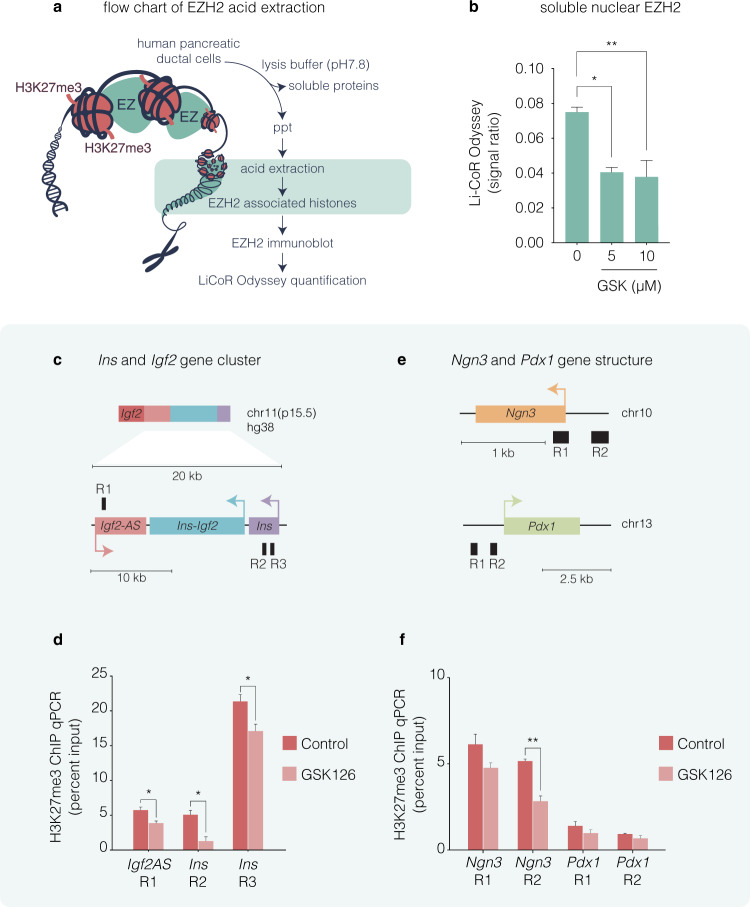
GSK126 influences the refractory H3K27me3 content on the insulin chromatin domain in human non-diabetic exocrine cells. **a** Flow chart of EZH2 acid extraction from human pancreatic ductal cells. Purification method of acid histone-associated protein fraction isolated from heterochromatin isolates derived from pancreatic ductal nuclei. Extraction of histone-binding EZH2 protein fraction from heterochromatin involve acid homogenisation and precipitation (ppt) with 5 M H_2_SO_4_. Isolated histone-binding proteins with EZH2 were fractionated and quantified using Li-COR Odyssey. **b** Dose-dependent increase of GSK126 (5 or 10 µM) for 48 h attenuates EZH2 in human pancreatic ductal cells. Quantification levels of EZH2 were calculated and adjusted to unmodified histone H3 using Li-COR Odyssey. The signal ratio was calculated as EZH2/overall H3. Vehicle control was DMSO. Ordinary one-way ANOVA was performed on Control vs GSK-126 (*P < 0.05, **P < 0.01, error bars are SEM, *n* = 3). **c** Insulin domain was assessed using amplimers that were specifically designed to distinguish promoter regions (R) of the *Ins* and *Igf2AS* genes. **d** Quantitative PCR analysis of DNA in chromatin immunoprecipitated (ChIP) with anti-H3K27me3 antibody. Vehicle control was DMSO. **e** DNA was assessed using amplimers that specifically recognise the promoter regions (R) of the *Ngn3* and *Pdx1* genes. **f** Quantitative PCR analysis of DNA in ChIP with anti-H3K27me3 antibody. Vehicle control was DMSO. Data represented as the mean Input signal against specific H3K27me3 abundance. Student’s *t*-test was performed on Control vs GSK-126 (*P < 0.05, **P < 0.01 error bars are SEM, *n* = 2)

The finding that the repressive H3K27me3 determinant is diminished by GSK126 and influences exocrine transcriptional competence supports a model in which EZH2 is tightly associated with chromatinization and gene silencing. Because the enzymatic transfer of methyl groups is catalysed to the specific K27 residue by histone methyltransferase activity we isolated EZH2 from human pancreatic ductal cells (Fig. 5a). Acidic purification of histone-binding proteins from ductal nuclei show GSK126 treatment at 5 and 10 µM diminishes EZH2 when compared to the recovery of overall histone H3 (Fig. 5b). These results suggest GSK126 can influence H3K27me3 by inhibiting EZH2 methyltransferase activity. To determine whether H3K27me3 content on the *Ins* chromatin domain (*Ins* and *Igf2AS*) could influence default suppression, we employed chromatin immunoprecipitation (ChIP) experiments from exocrine cells derived from non-diabetic donor (2) that were treated in the presence (5 µM GSK126) or absence (vehicle control, DMSO) of the EZH2 inhibitor. Chromatin-associated proteins were cross-linked and fractionated by sonication. DNA-associated chromatin was immunopurified using an antibody that specifically recognises H3K27me3. DNA was assessed by qPCR using amplimers (Table 3) that were specifically designed to detect human *Ins* chromatin domain genes (Fig. 5c). A marked reduction in H3K27me3 content was observed at the *Ins* and *Igf2AS* promoter (R1–R3) regions in exocrine cells treated with GSK126 (Fig. 5d). We also examined H3K27me3 promoter content on *Ngn3* and *Pdx1* promoters (Fig. 5e). In non-diabetic exocrine cells, H3K27me3 content on the *Ngn3* promoter of R1 was marginally altered whereas R2 was significantly reduced in cells treated with GSK126 (Fig. 5f). *Pdx1* DNA was recovered equally well from vehicle and GSK126-treated cells. These results suggest the chromatin content of the *Ins* chromatin domain is characterised by tight epigenetic control by H3K27me3 in human exocrine cells.

**Table 3:** Human primer sequences for ChIP

Primer name	Sequence
PDX1 PromR1 F	ACGTTTCTGCAAAGCTGTCTAGTT
PDX1 PromR1 R	GGCTTCAAACCATTCAGTAACTTC
PDX1 PromR2 F	TGGCTGTGAACAAACTTCATAAAT
PDX1 PromR2 R	CACCGTGGCTTAAAAGTTTCTATT
NGN3 PromR1 F	TTGCTCCTAGCCTATCTTTCCTTA
NGN3 PromR1 R	CTTTAGAATTCCTGGACCCTTCTC
NGN3 PromR2 F	CTTCTGGTCGCCAAGTTCAG
NGN3 PromR2 R	AGCAGATAAAGCGTGCCAAG
INS-IGF2 PromR1 F	GGGAACATAGAGAAAGAGGTCTCA
INS-IGF2 PromR1 R	AATTAATCTCAGCTTCCCCCTAAC
INS PromR2 F	TTTATAGTCTCAGAGCCCATCTCC
INS PromR2 R	CCCCTGGTTAAGACTCTAATGACC
INS PromR3 F	GACCTCATCTTCCGTGTGATCT
INS PromR3 R	CAGATGACACTATGGGGGTGAT

## Discussion

Taken together, we report that the action of the EZH2 inhibitor may facilitate the development of epigenetic compounds for β-cell regeneration. Not only are the data of direct clinical relevance, but our studies are also important to understand new mechanisms of gene regulation by pharmacological EZH2 inhibition.

There are significant limitations in the present study. First, the case is an isolated one, of a T1D child with hallmark islet damage and significant destruction of β-cells. Thus, it is unknown if the results will generalise. Nevertheless, it was possible to increase the expression of core β-cell determinants including insulin transcription in exocrine cells derived from two adult non-diabetic donors. Secondly, and equally important, it is unclear whether classic silencing of progenitor genes can be restored in long-standing diabetes. The ability to inhibit EZH2 and influence exocrine β-cell progenitor transcription to near non-diabetic mRNA levels suggests this is highly likely. In view of this evidence, this study is likely to influence research on using exocrine cells from the pancreas as a potential source to generate de novo insulin-producing β-cells and a new working hypothesis on β-cell plasticity. Furthermore, due consideration to the potential pharmacological interactions and unforeseen synergistic benefits warrant further study, which we predict will be an important area of study pertinent to exocrine regeneration and epigenetic research. While GSK126 is not licensed for diabetic mellitus the primary findings add to previous clinical reports demonstrating superior transcriptional reactivation by restoring histone content.^18^ Whether GSK126 treatment of exocrine cells restores the content of other histone modifications such as acetylation associated with the human insulin chromatin domain is unclear. Pharmacological EZH2 inhibition reduced trimethylation but failed to alter acetylation content at K27 of histone H3. The opportunity to investigate fresh exocrine tissue from a T1D donor and the availability of a pharmacological inhibitor of H3K27me3 have allowed better characterisation of the refractory nature of exocrine β-cell gene suppression.

Until now, the process of default suppression in the human pancreas was previously considered but challenging to study. The opportunity to investigate fresh tissue from donors, one diagnosed with childhood diabetes and the availability of a pharmacological compound that influence exocrine transcription, presumably by chromatinisation of the insulin gene cluster have allowed better characterisation of this still obscure aspect of exocrine competence. The stark reality of donor organ shortage limits widespread use of cadaveric pancreatic tissue in clinical epigenetics. This is complicated by the challenging nature of sourcing fresh tissue. While the results we have shown highlight the importance of human ex vivo research, we are also aware of the limitations in the study. Whether default transcriptional suppression reflect the epigenetic nature of genes in the pancreas and β-cell precursors needs to be confirmed in a larger series of studies.

**Fig. 6:**
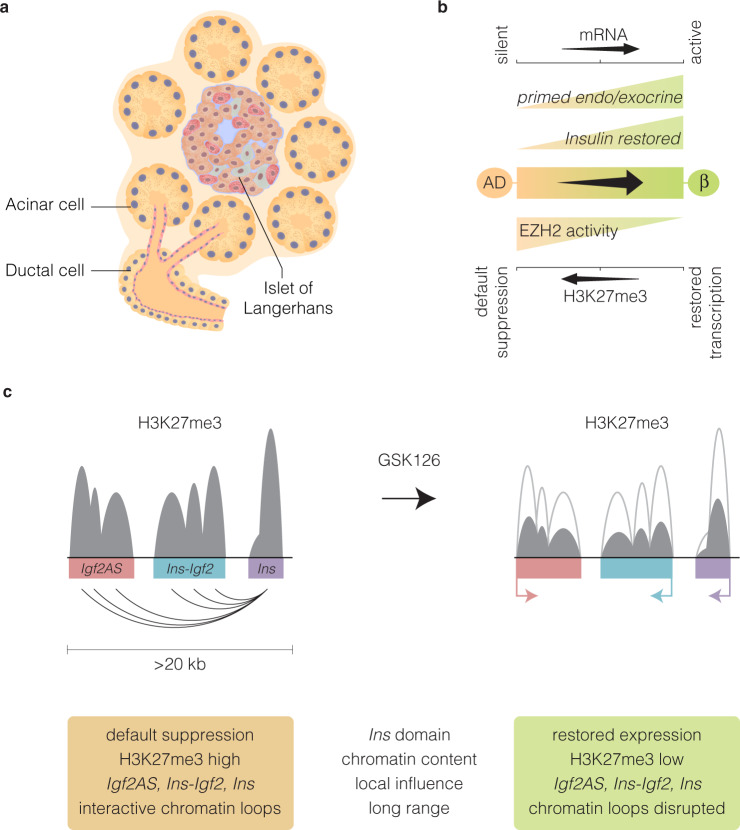
Schematic representation of pancreatic progenitor differentiation and the refractory influence of H3K27me3 content on the insulin chromatin domain in human exocrine cells. **a** Organisation of the human endocrine and exocrine pancreas showing the main pancreatic ductal tree connecting the acinar bundle. **b** The inability to influence transcriptional expression in the exocrine and endocrine pancreas is in accordance with default transcriptional suppression mediated by EZH2 dependent H3K27me3. Conversion of default repression state in the acinar and ductal (AD) cells is influenced by pharmacological inhibition of EZH2 by GSK126 to prime β-cell lineage regeneration and restore insulin expression. **c** Proposed model of the *Ins* chromatin domain in pancreatic cells. Default transcriptional suppression is characterised with H3K27me3 rich regions can influence chromatin conformation, and suppress the expression of *Ins, Igf2AS* and *Ins-Igf2* genes postulated by long-distance chromatinised-looping. We propose GSK126 attenuates EZH2 activity to influence local H3K27me3 chromatin content and long-distance interactions that function on the *Ins* chromatin domain and gene expression

Previous preclinical findings^14^ and those reported here, show that exocrine/endocrine Pdx1^+^ and endocrine Ngn3^+^ cells have the capacity to become insulin-producing cells. Because exocrine *Pdx1*, *Ngn3*, *Sox9, Sox11* and β-cell-specific markers *Nkx6.1, MafA*, and *Ins* gene expression are restored ex vivo we can predict that these changes are influencing β-cell neogenesis and that GSK126 targets refractory EZH2 mediated suppression (Fig. 6a). *MafA* is expressed exclusively in β-cells and regulates the expression of genes involved in insulin synthesis and secretion.^20^ *Nkx6.1* is a lineage allocation factor that drives progenitors toward a β-cell fate where its expression is restricted to β-cells and found to be required for proper insulin secretion.^21^ Importantly, we establish proof-of-concept exocrine barrier accounts for the inability to restore core progenitor genes that influence ex vivo plasticity despite total β-cell destruction (Fig. 6b). The insulin gene is part of a large chromatin domain associated with histone modifications that function to regulate gene expression in human islets.^15^ The assembly of specialised nucleosomal structures on H3K27me3 chromatin implicates the refractory capacity of the *Ins* domain—to suppress transcription more effectively—than conventional or open chromatin of exocrine cells not treated with GSK126 (Fig. 6c). Indeed, H3K27me3 content was shown to influence chromatin domain structures involving looping conformation to regulate *Igf2* gene expression.^22^ Although other factors involved in regulating insulin expression in T1D pancreata will need to be considered, including incomplete processing of proinsulin,^23^ our data emphasises refractory H3K27me3 content associated with exocrine competency and default transcriptional suppression.

The present report has interesting clinical implications for regenerative medicine within the context of T1D since it describes the first example of restoring transcription of the insulin gene, providing distinguishable evidence for β-cell regeneration and possibly neogenesis. Furthermore, this work reveals that human pancreatic progenitor cells retain their capacity to differentiate into neo-β-cells from the exocrine compartment that were derived from an insulin devoid T1D donor. These findings show that pharmacological alteration of cell fate decisions through the epigenetic induction of pancreatic progenitor genes holds promise for the treatment of T1D by promoting ex vivo β-cell regeneration through assisted epigenetic lineage reprogramming.

## Materials and methods

### Human samples

Rapid harvesting of cadaveric pancreatic tissues was obtained with informed consent from next of kin, from heart-beating, brain-dead donors, with research approval from the Human Research Ethics Committee at St Vincent’s Hospital, Melbourne. Pancreas from individuals without and with diabetes, islet, acinar and ductal samples were obtained as part of the research consented tissues through the National Islet Transplantation Programme (at Westmead Hospital, Sydney and the St Vincent’s Institute, Melbourne, Australia), HREC Protocol number: 011/04. The donor characteristics of islet cell donor isolations are presented in Table 1.

### Preparation and culture of isolated human pancreatic cells

Islets were purified by intraductal perfusion and digestion of the pancreases with collagenase AF-1.^24^ (SERVA/Nordmark, Germany) followed by purification using Ficoll density gradients.^25^ Purified islets, from low-density gradient fractions and acinar/ductal tissue, from high-density fractions, were cultured in Miami Media 1A (Mediatech/Corning 98–021, USA) supplemented with 2.5% human serum albumin (Australian Red Cross, Melbourne, VIC, Australia), in a 37 °C, 5% CO_2_ incubator.

### Gene expression analysis

Total RNA from human ex vivo pancreatic cells was isolated using TRIzol (Invitrogen) and RNeasy Kit (QIAGEN) including a DNase treatment. First-strand cDNA synthesis was performed using a high-capacity cDNA Reverse Transcription Kit (Applied Biosystems) according to the manufacturer’s instructions. cDNA primers were designed using oligoperfect designer (Thermo Fisher Scientific), as shown in Table 2. Briefly, quantitative RT-PCR analyses were undertaken using the PrecisionFast 2× qPCR Master Mix (Primerdesign) and primers using Applied Biosystems 7500 Fast Real-Time PCR System. Each qPCR reaction contained: 6.5 μl qPCR Master Mix, 0.5 μl of forward and reverse primers, 3.5 μl H_2_O and 2 μl of previously synthesised cDNA, diluted 1/20. Expression levels of specific genes were tested and normalised to 18s ribosomal RNA housekeeping gene.

### Acid extraction and quantitative immunoblotting

Modification of Histone H3 and histone-associated Ezh2 protein signals were quantified in human pancreatic ductal epithelial cells (AddexBio) by the LI-COR Odyssey assay. The cells were treated with 5 or 10 µM of GSK 126 (S7061, Selleckchem) for 48 h. Histones and their associated proteins were examined using an acid extraction and immunoblotting as described previously.^18^ Protein concentrations were determined using Coomassie Reagent (Sigma) with BSA as a standard. Equal amounts (3 µg) of acid extract were separated by Nu-PAGE (Invitrogen), transferred to a PVDF membrane (Immobilon-FL; Millipore) and then probed with antibodies against H3K27me3 (07–449, Millipore), H3K27ac (ab4729, Abcam), H3K9me3 (ab8898, Abcam), H3K9me2 (ab1220, Abcam), H3K4me3 (39159, Active Motif), Ezh2 (#4905, Cell Signaling Technology), and total histone H3 (#14269, Cell Signaling Technology). Protein blotting signals were quantified by an infra-red imaging system (Odyssey; LI-COR). Modification of Histone H3 and histone-associated Ezh2 signals were quantified using total histone H3 signal as a loading control.

### Chromatin immunoprecipitation

Chromatin immunoprecipitation assays in human exocrine cells were performed previously described.^26,27^ Cells were fixed for 10 min with 1% formaldehyde and quenched for 10 min with glycine (0.125 M) solution. Fixed cells were resuspended in sodium dodecyl (lauryl) sulfate (SDS) lysis buffer (1% SDS, 10 mM EDTA, 50 mM Tris-HCl pH 8.1) including a protease inhibitor cocktail (Roche Diagnostics GmBH, Mannheim, Germany) and homogenised followed by incubation on ice for 5 min. Soluble samples were sonicated to 200–600 bp and chromatin was resuspended in ChIP Dilution Buffer (0.01% SDS, 1.1% Triton X-100, 1.2 mM EDTA, 16.7 mM Tris-HCl pH 8.0, and 167 mM NaCl) and 20 µl of Dynabeads^®^ Protein A (Invitrogen, Carlsbad, CA, USA) was added and pre-cleared. H3K27me3 antibody was used for immunoprecipitation of chromatin and incubated overnight at 4˚C as previously described.^28^ Immunoprecipitated DNA were collected by magnetic isolation, washed low salt followed by high salt buffers and eluted with 0.1 M NaHCO_3_ with 1% SDS. Protein-DNA cross-links were reversed by adding Proteinase K (Sigma, St. Louis, MO, USA) and incubation at 62 °C for 2 h. DNA was recovered using a Qiagen MinElute column (Qiagen Inc., Valencia, CA, USA). H3K27me3 content at the promoters of the *INS*, *INS-IGF2*, *NGN3* and *PDX1* genes were assessed by qPCR using primers designed from the integrative ENCODE resource.^29^ ChIP primers are shown in Table 3.

### Immunohistochemistry

Insulin and glucagon localisation in human islets were assessed using paraffin sections (5 μm thickness) of human pancreas tissue fixed in 10% neutral-buffered formalin and stained with hematoxylin and eosin (H&E) or prepared for immunohistochemistry. Insulin and glucagon were detected using Guinea Pig anti-insulin (1/100, DAKO) or mouse anti-glucagon (1/50) mAbs (polyclonal Abs, Sigma-Aldrich).

### Ex vivo stimulation of human pancreatic progenitors with GSK126

Pharmacological inhibition of EZH2, human pancreatic exocrine cells were kept untreated or stimulated with 10 μM GSK-126 (S7061, Selleckchem) at a cell density of 1 × 10^5^ per well for 24 h. After 24 h of treatment, fresh Miami Media was added to the cells, which were treated again with 10 μΜ GSK-126 and cultured for a further 24 h. All cell incubations were performed in Miami Media 1A (Mediatech/Corning 98-021, USA) supplemented with 2.5% human serum albumin (Australian Red Cross, Melbourne, VIC, Australia), in a cell culture incubator at 37 °C in an atmosphere of 5% CO_2_ for 48 h using non-treated six-well culture plates (Corning).
